# Low cardiac output as physiological phenomenon in hibernating, free-ranging Scandinavian brown bears (*Ursus arctos*) – an observational study

**DOI:** 10.1186/1476-7120-12-36

**Published:** 2014-09-16

**Authors:** Peter Godsk Jørgensen, Jon Arnemo, Jon E Swenson, Jan S Jensen, Søren Galatius, Ole Frøbert

**Affiliations:** Department of Cardiology, University of Copenhagen, Gentofte Hospital, Copenhagen, Denmark; Department of Forestry and Wildlife Management, Faculty of Applied Ecology and Agricultural Sciences, Hedmark College, Campus Evenstad, Elverum, NO-2418 Norway; Department of Wildlife, Fish and Environmental Studies, Faculty of Forest Sciences, Swedish University of Agricultural Sciences, SE-901 83 Umeå, Sweden; Department of Ecology and Natural Resource Management, Norwegian University of Life Sciences, NO-1528 Ås, Norway; Norwegian Institute for Nature Research, NO-7485 Trondheim, Norway; Department of Cardiology, Örebro University Hospital, Örebro, Sweden

**Keywords:** Animal model cardiovascular disease, Acute cardiac care, Thrombosis, Echocardiography

## Abstract

**Background:**

Despite 5-7 months of physical inactivity during hibernation, brown bears (Ursus arctos) are able to cope with physiological conditions that would be detrimental to humans. During hibernation, the tissue metabolic demands fall to 25% of the active state. Our objective was to assess cardiac function associated with metabolic depression in the hibernating vs. active states in free-ranging Scandinavian brown bears.

**Methods:**

We performed echocardiography on seven free-ranging brown bears in Dalarna, Sweden, anesthetized with medetomidine-zolazepam-tiletamine-ketamine during winter hibernation in February 2013 and with medetomidine-zolazepam-tiletamine during active state in June 2013. We measured cardiac output noninvasively using estimates of hemodynamics obtained by pulsed wave Doppler echocardiography and 2D imaging. Comparisons were made using paired T-tests.

**Results:**

During hibernation, all hemodynamic indices were significantly decreased (hibernating vs. active state): mean heart rate was 26.0 (standard deviation (SD): 5.6) beats per min vs. 75.0 (SD: 17.1) per min (P = 0.002), mean stroke volume 32.3 (SD: 5.2) ml vs. 47.1 (SD: 7.9) ml (P = 0.008), mean cardiac output 0.86 (SD: 0.31) l/min vs. 3.54 (SD: 1.04) l/min (P = 0.003), and mean cardiac index 0.63 (SD: 0.21) l/min/kg vs. 2.45 (SD: 0.52) l/min/ m^2^ (P < 0.001). Spontaneous echo contrast was present in all cardiac chambers in all seven bears during hibernation, despite the absence of atrial arrhythmias and valvular disease.

**Conclusion:**

Free-ranging brown bears demonstrate hemodynamics comparable to humans during active state, whereas during hibernation, we documented extremely low-flow hemodynamics. Understanding these physiological changes in bears may help to gain insight into the mechanisms of cardiogenic shock and heart failure in humans.

**Electronic supplementary material:**

The online version of this article (doi:10.1186/1476-7120-12-36) contains supplementary material, which is available to authorized users.

## Background

Brown bears (*Ursus arctos*) have unique physiological adaptations to deal with the annual hibernation period lasting 5-7 months, in which they do not eat, drink, defecate, or urinate and display minimal physical activity [1]. These adaptations protect the bears from the detrimental effects of inactivity and, hence, they avoid the loss of muscle and bone mass [2–5], decubitus ulcers, and the deterioration of cardiac function [6–9]. During this period the bears’ oxygen demand is reduced to 25% of the active state [10] and cardiac adaptations, including profound bradycardia and low cardiac output are induced to optimize energy conservation during the long period of low metabolism. Whereas bed rest and sitting still for longer periods of time predispose humans to thromboembolism [11], this is apparently not the case in the brown bear. Clearly, the identification of mechanisms responsible for these adaptations could have substantial applications for various areas of human medicine including intensive care medicine and in prevention of thromboembolism.

In humans, spontaneous echo contrast (SEC) in the echocardiogram is the presence of swirling echo-dense shadows in the cardiac chambers and large vessels. SEC occurs in areas of low blood flow, and is caused by the aggregation of red blood cells and plasma proteins [12, 13]. The presence of SEC is strongly associated with cardiac pathophysiology in humans and predisposes to thromboembolic events [13–18].

Reduced cardiac output during hibernation has been described in bears previously [7, 8] but a more detailed description of the cardiac functional adaptations is warranted for a deeper understanding of the physiology of the brown bears. In addition, the previous studies were conducted in captive bears with the risk of introducing bias caused by human interaction and/or presence during hibernation [19]. Hence, the aim of this study was to provide a detailed documentation of the cardiac function and intracardiac blood flow pattern in the hibernating and in the active free-ranging brown bears.

## Methods

### Material

We assessed the hemodynamics of seven subadult hibernating brown bears in Dalarna, Sweden in February 2013. These bears had received GPS collars previously, which allowed us to locate them in their dens. The bears were immobilized using a mixture of medetomidine, zolazepam, tiletamine and ketamine [20]. The same bears, in the active state, were immobilized in June 2013, where they were darted from a helicopter with medetomidine, zolazepam and tiletamine at 2-4 times the winter dose [20]. The study was approved by the Swedish Ethical Committee on Animal Research (C212/9) and the procedure was in compliance with Swedish laws and regulations.

### Echocardiography

We performed echocardiography in the field with the bear in a left lateral recumbency using a Phillips CX50 with an S51 probe. We used second harmonic imaging and the obtained images included 2D, MMode, and pulsed and continuous wave spectral Doppler echocardiography. We estimated stroke volume using the formula.


where SV is the stroke volume (ml), D is the left ventricular outflow tract (LVOT) diameter (cm), measured in the parasternal long axis view, and TVI is the time velocity integral (cm) of the pulsed wave spectral Doppler in the LVOT after careful alignment of the marker in the direction of the blood flow. To minimize the influence of measurement error of the LVOT diameter, we used each bear’s mean value of LVOT diameter measured during hibernation and active state to calculate SV. Cardiac output (l/min) was calculated using the formula.


where HR (beats/s) is the mean heart rate obtained from 6 heart beats. We calculated the cardiac index (CI) (l/min/m^2^) using an estimation of body surface area (BSA) (m^2^) as previously described [21].


We measured ejection time and time to peak velocity from the pulsed wave spectral Doppler placed in LVOT as above. We obtained cardiac intervals with the pulsed wave spectral Doppler placed between mitral leaflets during diastole. Isovolumetric relaxation time (IVRT) + Isovolumetric contraction time (IVCT) was calculated as end of A wave to start of E wave *minus* ejection time and Tei index was calculated as (IVRT + IVCT)/ejection time.

Echocardiograms, post-processing, and assessment of SEC were performed by an experienced echocardiographer (PGJ).

### Statistics

The values are presented as mean ± 1 standard deviation (SD). Because of the small number of bears, normal distribution of values was difficult to assume. However, the results of the paired T-tests were chosen to be shown with the limitation that normal distribution of data might not be fulfilled. The calculations were made using the statistical software package ‘R’ , version 3.0.1 (64 bit) (R Project for Statistical Computing, http://www.R-project.org).

## Results and discussion

In total, we examined the same seven bears in the field in both February and June. Echocardiograms were only obtained in six of the bears (4 females and 2 males) in June 2013. During hibernation the bears did not differ significantly from the active state in body mass (50.3 kg vs. 54.3 kg; P = 0.50).

We found no valve disorders or arrhythmias, during the echocardiographic examinations.

**Figure 1:**
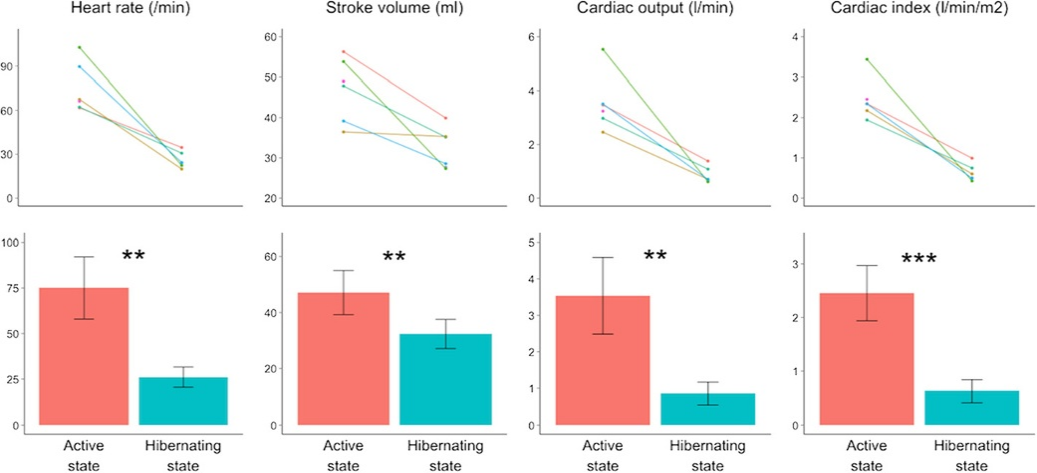
**Differences in heart rate, stroke volume, cardiac output, and cardiac index between the hibernating and active states of free-ranging brown bears.** First row shows repeated measurements from each bear, second row shows mean values and standard deviations. Levels of significance: *P < 0.05, **P < 0.01 and ***P < 0.001. During hibernation, all assessed hemodynamic parameters were significantly decreased as an adaptation to low energy demands. During hibernation, the cardiac index, which is cardiac output related to body size, had a level that would imply very severe cardiogenic shock in humans and, thus, would be largely incompatible with life.

Flow indices for each bear and mean values are found in Figure 1. During hibernation, all flow indices were reduced compared with the active state (hibernating vs. active state): heart rate was reduced to 35% (26.0 (±5.6) beats per min vs. 75.0 (±17.1) beats per min (P = 0.002)); stroke volume to 69% (32.3 (±5.2) ml vs. 47.1 (±7.9) ml (P = 0.008)); cardiac output to 24% (0.86 (±0.31) l/min vs. 3.54 (±1.04) l/min (P = 0.003)) and cardiac index to 26% (0.63 (±0.21) l/min/m^2^ vs. 2.45 (±0.52) l/min/m^2^ (P < 0.001)).

Pulsed wave Doppler-derived measurements of cardiac intervals are found in Table 1. Both systolic and diastolic time intervals were increased during hibernation. However, although ejection time was increased by 30% and time to peak velocity was increased by 70%, the most prominent increase was in diastolic filling time, which increased by a factor 3.9. Likewise, the Tei index was increased by 66% during hibernation, indicating relatively longer periods of isovolumetric phases.

**Table 1:** **Cardiac time intervals measured by pulsed wave doppler echocardiography**

	Active state	Hibernating state	P-value
Ejection time (ms)	200 (±18.6)	256 (±16.9)	0.005
Diastolic filling time (ms)	571 (±103)	2217 (±858)	0.02
IVRT + IVCT (ms)	363 (±30)	583 (±61)	0.006
Tei index	0.77 (±0.10)	1.28 (±0.29)	0.07

IVRT = isovolumetric ventricular relaxation time, IVCT = isovolumetric ventricular contraction time.

**Figure 2:**
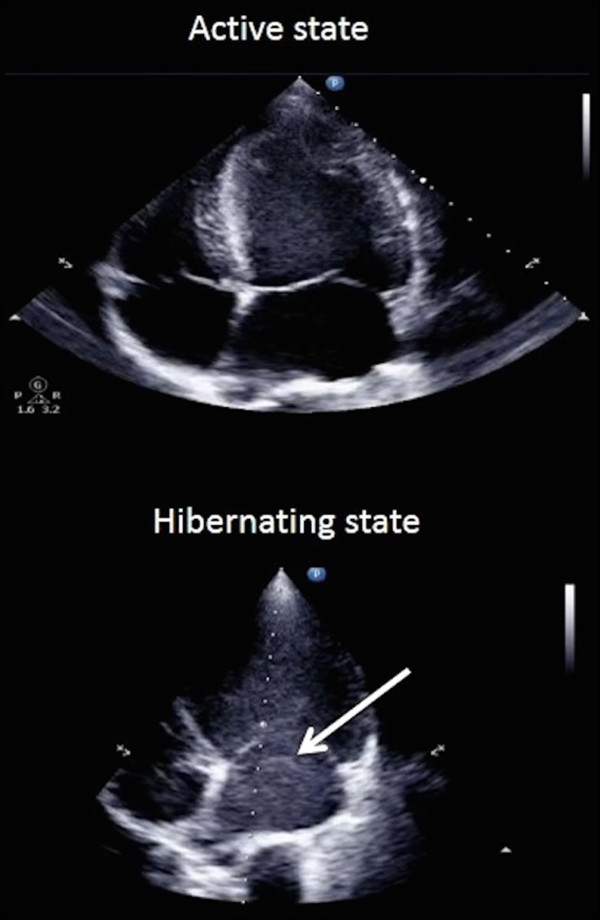
**Comparison of 2D echocardiographic images from the hibernating and active states of free-ranging brown bears.** An arrow indicates the presence of spontaneous echo contrast as echo-dense shadows during hibernation. Also see Additional files 1 and 2.

SEC was present as swirling echo-dense structures in all four chambers of all seven bears examined in the hibernating state in February 2013. However, there were no signs of SEC in the bears when examined during their active state in June 2013 (Figure 2 and Additional files 1 and 2).

In this study, we describe the pattern of extreme low flow hemodynamics in hibernating brown bears. Although all measured flow indices were significantly reduced during hibernation, the difference was most pronounced for CO, with more than a four-fold decrease during hibernation. In addition, even CI, which takes in to account both differences in HR, SV and animal size and provides the most accurate assessment of changes in hemodynamics, was increased almost four-fold during the active state. The low flow hemodynamics were reflected by the presence of SEC in all cardiac chambers.

### Low blood flow hemodynamics and metabolism: potential perspectives for human medicine

All assessed hemodynamic parameters (SV, CO, CI) were significantly reduced during hibernation, indicating adaptation to low energy demands. The magnitude of this metabolic change can be illustrated by comparing to human values, where CI is considered normal when between 2.6 and 4.2 l/min/m^2^ and cardiogenic shock may be present when the CI drops below 1.8 l/min/m^2^ [22]. Thus, the CI during the bears’ active state was within the normal range for humans, but during hibernation it was reduced to an extent that is largely incompatible with life in humans. Whereas treatment of cardiogenic shock is traditionally centered on reestablishing adequate tissue perfusion by improving cardiac output [22], there is less focus on minimizing tissue damage by decreasing the metabolic demands in the peripheral tissues.

In some clinical settings, such as after cardiac arrest and during cardiac surgery, induction of slight hypothermia is an established treatment. In these cases, hypothermia acts by suppressing metabolism and is thought to prevent primarily brain damage during and after periods of impaired end-organ oxygen delivery [23]. However, according to a recent study that examined the cardiac effects of therapeutic hypothermia as measured with echocardiography; [24] the isolated hypothermic effect in pigs is different from the adaptations in the hypothermic, hibernating bear. In hypothermic pigs like in hibernating bears, the cardiac output was slightly decreased and the duration of the systole was increased with increased ejection time. In hypothermic pigs, however, heart rate and duration of the diastole were slightly reduced while, in the hibernating bear, heart rate was about one third of the active state and diastolic filling time increased almost 4 fold. Hence, our data suggest that the cardiac adaptations evident during hibernation are different from that obtained by mere hypothermic metabolic suppression. This is supported by a recent study, showing that metabolic suppression in hibernating American black bears (Ursus americanus) is independent of lowered body temperature [10].

A substance derived from the serum of hibernators with the ability to induce a hibernation-like state in nonhibernators may exist [25, 26]. This so-called ‘hibernation-inducing trigger’ has also been identified in hibernating bear serum, and there is evidence that exposing rabbits’ hearts to hibernating bear serum prior to cardioplegia reduces ischemia-reperfusion injury [27], thus indicating that bear serum has the capability to reduce myocardial oxygen demands similar to the hibernating myocardium. Other substances that can induce hibernation-like states in nonhibernating animals also exist. Exposing mice to hydrogen sulfide (H_2_S) induces a ‘suspended animation-like state’ , with decreased metabolism, core body temperature [28], and cardiac and respiratory effects very similar to those found in the hibernating bear [29].

### Spontaneous echo contrast – evidence of cause and consequences

Blood is usually echo-lucent in the cardiac chambers. Occasionally blood appears as a swirling haze of echo-dense structures, which is caused by the aggregation of red blood cells and plasma proteins [12, 13] and is exclusively seen under conditions of low blood flow, or stasis, with low shear stress. Dense SEC has even been suggested to be a transition state in the formation of fibrin-rich, red thrombi [30, 31]. In humans, conditions predisposing to low flow and/or stasis conditions include atrial fibrillation, mitral stenosis, and dilated cardiomyopathy. In all three conditions, the presence of SEC is an ominous sign of risk for future thromboembolic disease and is used to guide clinical decision-making on a daily basis [13–18]. Though the mechanisms of SEC may be species specific (making it difficult to directly compare the species), our findings suggest that the mechanisms underlying the formation of SEC persist and remain unaltered during the brown bears’ hibernation period. This, in turn, implies that the adaptations in blood coagulation during hibernation might involve hematological mechanisms not essential for SEC formation.

### Clinical perspectives

The hibernating bear model reminds the clinicians that metabolism is not a fixed entity, but that it is perpetually adjusting to the prevailing circumstances. Careful adjustment of treatment is advised when using medications that modify metabolism in critically ill patients. This is, for instance, the case with vasoactive agents, like catecholeamines, that in addition to their vasoactive effect, increase organ metabolism, and hence oxygen consumption [32]. The existence of substances that can induce a hibernation-like state in nonhibernating animals indicates that low-metabolism conditions can be medically induced in other species and opens for more research on possible applications for humans.

The finding of SEC in all cardiac chambers in the hibernating bear, which is apparently free from thromboembolic events, suggests that the increased risk of thromboembolic events in humans with SEC is not consequence of SEC itself, but may merely be a marker of the severity of the underlying disease.

### Strengths and limitations

The free-ranging brown bear used in the present study is a strong model to understand biomimicry, the imitation of models in nature for the purpose of solving complex human problems, because bears are undisturbed by human interaction and/or presence. This allows for the study of bears in their natural habitat, without potential bias introduced by captivity, such as audible, visual, and olfactory stimuli and unnatural food availability. Our model, however, is limited by the fact that the bears were immobilized using anesthetics, which might induce changes in hemodynamic parameters [8]. Our finding of reduced SV differs from previous studies on unanesthetized bears and thus the measurements might have been affected by the anesthesia, differences in methods used to calculate SV, or a combination of both. However, we consider this unlikely, because of the lower doses of anesthetics used in winter compared with summer captures. Moreover, the differences we found in HR, CO, and CI agree with earlier studies [8]. SEC was only present in the hibernating state and with reference to doses. We find no reason to believe that the described low-flow conditions and the presence of SEC were caused solely by the use of anesthetics.

A more comprehensive echocardiographic examination would have been preferable. However, taken into account the circumstances of especially the winter examinations – the sub-zero temperatures, limited time-frame for the echocardiographic examination and the need to limit the area that is shaved on the bear – the number of measurements to be collected in a reproducible manner is limited.

## Conclusion

Low blood flow hemodynamics and SEC are normal physiological phenomena in free-ranging Scandinavian brown bears in response to low energy demands during hibernation. Identification of the mechanisms responsible for the lowered metabolic rate and decreased hemodynamic indices could have implications for human medicine, including treatment of cardiogenic shock, heart failure and prevention of thromboembolism.

## Electronic supplementary material

Additional file 1: **2D echocardiographic images from the hibernating state of free-ranging brown bears.** The presence of spontaneous echo contrast as swirling, echo-dense shadows is easily recognized in the left atrium and ventricle. The blood is almost entirely halted during parts of the prolonged diastole. (MP4 4 MB)

Additional file 2: **2D echocardiographic images from the active state of free-ranging brown bears.** There is no evidence of spontaneous echo contrast in neither left atrium nor ventricle. (MP4 1 MB)

## Abbreviations

BSA: Body surface area
CI: Cardiac index
CO: Cardiac output
HR: Heart rate
IVCT: Isovolumetric contraction time
IVRT: Isovolumetric relaxation time
LVOT: Left ventricular outflow tract
SD: Standard deviation
SEC: Spontaneous echo contrast
SV: Stroke volume
TVI: Time velocity integral.
